# 

**Published:** 1842-07-23

**Authors:** 


**CONTENTS OF No. 30.**

BIBLIOGRAPHICAL NOTICE: Of Vital Action in Animals, and of the Influence of the Atmosphere. By J. Liebig, - - - - 465

ANALECTA: Dr. Hunt's Case of General Hæmorrhagic Tendency; with Remarks by Dr. Reynell Coates, - - - - - 472

Abstract of Professor Heim's Paper on the identity of Small-Pox and Cow-Pock, - - - - - - - - 476

Dr. J. B. S. Jackson on the Comparative Frequency of Tuberculous Disease, - - - - - - - 477

Dr. Rowbotham on the Effects of a Diet of Fruit and Saccharine Food, - - - - - - - - - 479

Dr. M. Marotte on Emetics in confirmed Croup, - - 480

